# Identification and Biochemical Characterization of a Novel Hormone-Sensitive Lipase Family Esterase Est19 from the Antarctic Bacterium *Pseudomonas* sp. E2-15

**DOI:** 10.3390/biom11111552

**Published:** 2021-10-20

**Authors:** Xiaoyu Liu, Mingyang Zhou, Shu Xing, Tao Wu, Hailun He, John Kevin Bielicki, Jianbin Chen

**Affiliations:** 1 School of Chemistry and Chemical Engineering, Qilu University of Technology (Shandong Academy of Sciences), Jinan 250353, China; liuxiaoyu27@126.com (X.L.); xingshu@qlu.edu.cn (S.X.); 17806022232@163.com (T.W.); 2 State Key Laboratory of Medical Genetics, School of Life Sciences, Central South University, Changsha 410013, China; helenhe@csu.edu.cn; 3 Lawrence Berkeley National Laboratory, Berkeley, CA 94720, USA; jkbielicki@hotmail.com

**Keywords:** esterase, Antarctic, HSL family, GESAG motif

## Abstract

Esterases represent an important class of enzymes with a wide variety of industrial applications. A novel hormone-sensitive lipase (HSL) family esterase, Est19, from the Antarctic bacterium *Pseudomonas* sp. E2-15 is identified, cloned, and expressed. The enzyme possesses a GESAG motif containing an active serine (S) located within a highly conserved catalytic triad of Ser^155^, Asp^253^, and His^282^ residues. The catalytic efficiency (*k*_cat_/*K*_m_) of Est19 for the *p*NPC6 substrate is 148.68 s^−1^mM^−1^ at 40 °C. Replacing Glu^154^ juxtaposed to the critical catalytic serine with Asp (E154→D substitution) reduced the activity and catalytic efficiency of the enzyme two-fold, with little change in the substrate affinity. The wild-type enzyme retained near complete activity over a temperature range of 10–60 °C, while ~50% of its activity was retained at 0 °C. A phylogenetic analysis suggested that Est19 and its homologs may represent a new subfamily of HSL. The thermal stability and stereo-specificity suggest that the Est19 esterase may be useful for cold and chiral catalyses.

## 1. Introduction

Lipolytic enzymes are widely distributed in nature (i.e., animals, plants, and microorganisms), and a relatively large number has been isolated from metagenomes and bacteria [1,2,3,4,5,6,7,8,9,10]. The latter includes enzymes with different biological activities isolated from genera such as *Pseudomonas* [1], *Psychrobacter* [5], *Salinisphaera* [9], *Serratia* [11], *Burkholderia* [12], and *Staphylococcus* [13].

Lipolytic enzymes include two major classes: 1). Carboxylesterases (EC 3.1.1.1, carboxylester hydrolases, ‘true’ esterase) which hydrolyze ester-containing molecules, and preferentially hydrolyze ‘simple’ esters and usually only triglycerides composed of fatty acids shorter than C6, and 2). Lipases (EC 3.1.1.3, triacylglycerol hydrolases, ‘true’ lipase) which catalyze the hydrolysis of water-insoluble substrates, such as triglycerides composed of long-chain fatty acids [14,15,16]. These enzymes are members of the α/β hydrolase superfamily [17].

Esterases can catalyze the hydrolysis of ester bonds or the synthesis of ester linkages [16]. In recent years, esterases that retain activity over a broad range of temperatures have been identified and used for industrial applications. Enzymes with activity at low temperatures can hydrolyze heat-labile compounds; thus, avoiding unnecessary losses when heating the reactants [18]. Esterases with broad substrate specificity and excellent stereo- and regional selectivity have also been described [19]. These enzymes are widely used in food, cosmetic, and pharmaceutical industries, papermaking, and environmental management [20].

Bacterial lipolytic enzymes have been divided into eight families by Arpigny and Jaeger [15]. The fourth family, namely, the hormone-sensitive lipase (HSL) family, has a high amino acid sequence similarity to human HSL [15]. Compared to lipolytic enzymes of other families, the HSL family has a higher stability and wider substrate specificity. The active site of the HSL family members consists of a catalytic triad of Ser-His-Asp residues. For most esterases and lipases belonging to the HSL family, the catalytic Ser is located in a highly conserved motif (G-X-S-X-G) [21,22], where the amino acid at position X can vary. Bacterial HSL have been classified into two subfamilies: one containing a GDSAG motif and the other with a GTSAG motif [23]. Examples of family members include esterases with the following motifs: GDSAG, GDSGG, GDSSG, GSSAG, GQSAG, GESGG, GTSAG, GTSTG, GTSGG, and GSSGG.

Recently, we found a novel esterase gene *est19* from the genome of *Pseudomonas* sp. E2-15 isolated from Antarctic soil [24]. In the present study, we cloned the *est19* gene and overexpressed it in *E. coli*. The purified esterase protein was then characterized. A sequence analysis indicated that Est19 belongs to the HSL family, and that it possesses a unique GESAG catalytic motif.

## 2. Materials and Methods

### 2.1. Bacterial Strains and Expression Vector

The *est19* gene was identified from the genome sequence of *Pseudomonas* sp. E2-15, previously isolated from a soil sample collected on King George Island, Antarctica [24]. *E. coli* BL21 (DE3) and the pET-22b expression vector (Novagen, USA) were used for recombinant protein expression.

### 2.2. Sequence and Phylogenetic Analyses

Signal sequence prediction was carried out using SignalP server 5.0 (http://www.cbs.dtu.dk/services/SignalP/, accessed on 8 July 2020) [25]. The esterase family that the protein belongs to was confirmed using the ESTHER database (http://bioweb.ensam.inra.fr/esther, accessed on 8 July 2020) [26]. Reference amino acid sequences were retrieved from NCBI and PDB databases by protein BLAST (Basic Local Alignment Search Tool). Multiple-sequence alignment was performed by ClustalX [27] and DNAMAN. The phylogenetic tree was constructed by MEGA version 7.0.21 [28] using the Maximum Likelihood method based on the JTT matrix-based model. Sequence of the *est19* gene was deposited in the GenBank with the accession number MT761613.

### 2.3. Cloning, Overexpression and Purification of Recombinant Est19 and Its Mutants

The primers *est19F* (TTAAGAAGGAGATATACATatggcgcacaccccctggcctgcc) and *est19R* (GGTGGTGGTGGTGGTGCTCGAGcgaagactttccacctgtgta) were used to amplify the *est19* gene from the genome of *P.* sp. E2-15. The nucleotides pairing with the vector sequence were showed in uppercase letters, while the esterase gene sequence in lowercase letters.

In order to explore the role of Glu^154^ in the catalytic motif GESAG, we designed an E154D point substitution. The primers *EDmutF* (GTGATCATTGTTGGT***GAT***TCAGCGGGCGGCCATCT) and *EDmutR* (AGATGGCCGCCCGCTGA***ATC***ACCAACAATGATCAC) were used to construct the E154D variant by site-directed mutagenesis. The codon encoding Asp^154^ was in italics and bold.

The purified PCR product was ligated with the linearized pET-22b digested with the restriction enzymes *Nde*I and *Hind*III (Thermo Fisher Scientific, Waltham, MA, USA) using NovoRec^®^ plus One-step PCR Cloning Kit (Novoprotein, Shanghai, China). The generated pET22b-*est19* or pET22b-*est19*^E154D^ vector constructs were transformed into *E. coli* BL21 (DE3) cells. The inserted genes were verified by sequence analysis. The recombinant plasmids encoded a C-terminal His tag.

For expression, *E. coli* BL21 (DE3) cells carrying the recombinant plasmid were cultured in Luria Bertani (LB) broth at 37 °C, 200 rpm supplemented with ampicillin (100 μg/mL). Isopropyl-β-D-thiogalactopyranoside (IPTG, Merck, Darmstadt, Germany) was added at a final concentration of 0.05 mM when the OD_600_ was 0.6–0.8, and the cells were further cultivated at 16 °C for 24 h.

For purification, the induced cells were harvested by centrifuge at 4 °C with 10,000× *g* for 10 min and then washed 2 times with lysis buffer (50 mM Tris-HCl, 100 mM NaCl, pH 8.0). The cells were disrupted by sonication and then centrifuged at 11,000× *g* for 30 min at 4 °C. The supernatant was purified with Ni-Sepharose Fast Flow resin (GE Healthcare, Boston, MA, USA). Elution buffer (50 mM Tris-HCl, 100 mM NaCl, 50 mM or 250 mM imidazole, pH 8.0) was used to elute the protein. The purified protein was desalted by dialysis against 50 mM Tris-HCl (pH 8.0) to remove imidazole and NaCl. The sequencing of N-termini was carried out in Biotech-Pack Company (Beijing, China).

### 2.4. Esterase Assay

The concentration of esterase was measured by the method of Bradford. Esterase activity was measured as described by Li et al. [23]. The substrates *p*-nitrophenyl esters (*p*NPs; acetate, C2; butyrate, C4; caproate, C6; caprylate, C8; caprate, C10; laurate, C12; palmitate, C16) were prepared as 10 mM in 2-propanol, respectively. The standard reaction system contained 0.02 mL of 10 mM *p*NPC6, 0.96 mL of 50 mM Tris-HCl buffer (pH 8.0), and 0.02 mL of enzyme. After incubation at 40 °C for 10 min, the reaction was stopped by addition of 0.1 mL of 20% SDS (*w*/*v*). The absorbance at 405 nm was measured for quantifying the *p*-nitrophenol released. One unit (U) of enzyme was defined as the amount of enzyme required to release 1 μmol *p*-nitrophenol per minute. The blank control was created by substituting enzyme with 0.02 mL of buffer using the same reaction system.

Substrate specificity was determined with *p*NP esters (C2, C4, C6, C8, C10, C12, and C16). The optimum temperature was assessed from 0 to 90 °C with an interval of 10 °C and the optimum pH was assessed from pH 4 to 11 using Britton–Robinson buffer with an interval of 1 or 0.5 at 40 °C.

Triacylglycerol hydrolase activity was determined by titration method as described by Li et al. with modification using tributyrin, tricaprylin, and trilaurin as substrates [29]. Briefly, the triacylglycerol emulsions with concentration of 10 mM were prepared in 2.5 mM Tris-HCl (pH 7.0) containing 100 mM NaCl and 1% (*w*/*v*) gum arabic by mechanical stirring. The emulsion pH was adjusted to 7.00 by adding 5 mM NaOH. The reaction system contained 20 mL triacylglycerol emulsion and 0.5 mL enzyme. The same reaction system with 0.5 mL buffer instead of enzyme was used as the blank control. Reaction was stopped by the addition of 5 mL ethanol after incubation at 40 °C for 30 min. The system pH was adjusted to 7.00 by adding 5 mM NaOH. The released fatty acids were measured by the amount of NaOH added. One unit (U) was defined as the amount of enzyme required to release 1 μmol of fatty acid per minute.

The hydrolysis activity to chiral esters was determined with (1S)-menthyl acetate and (1R)-menthyl acetate, methyl (S)-3-hydroxybutyrate and methyl (R)- 3-hydroxybutyrate, D-pantolactone and L-pantolactone, methyl (S)-3-hydroxy-2- methylpropionate and methyl (R)-3-hydroxy-2-methylpropionate, D-alanine methyl ester and L-alanine methyl ester, D-tryptophan methyl ester and L- tryptophan methyl ester, methyl L-lactate and methyl D-lactate. The chiral esters were prepared as 143 mM in acetonitrile. The reaction system contained 0.2 mL of 5 mM EPPS buffer (pH 8.0) with 0.455 mM phenol red, 0.015 mL 143 mM esters, and 0.01 mL of 0.5 mg/mL Est19 enzyme. The difference of absorbance at 550 nm or the color before and after incubation at 40 °C for 4 h was used to determine the activity. The blank control used the same reaction system with 0.01 mL of 50 mM Tris-HCl buffer (pH 8.0) instead of enzyme.

### 2.5. Effect of NaCl Concentration, Inhibitors, Metal Ions, and Organic Solvents on the Esterase Activity

The effect of NaCl on enzymatic activity was measured using concentrations ranging from 0 to 3.5 M. The effects of metal ions (Li^+^, K^+^, Mg^2+^, Ca^2+^, Mn^2+^, Fe^2+^, Co^2+^, Ni^2+^, Cu^2+^, and Zn^2+^) and potential inhibitors (PMSF, EDTA, DTT, SDS, and TWEEN 80) on Est19 activity were measured with addition of 1 and 10 M final concentration of each agent in the reaction mixture. The effect of organic solvents (methanol, formaldehyde solution, ethanol, acetonitrile, acetone, isopropyl alcohol, and dimethyl sulfoxide) on enzymatic activity was measured at concentrations of 20% and 40% (*v*/*v*).

### 2.6. Kinetic Parameters of Est19 and Its Mutant

Enzyme kinetic assays were carried out in 50 mM Tris-HCl buffer (pH 8.0) at 40 °C, using *p*NPC6 as a substrate at 0.01 to 4.0 mM final concentrations. Kinetic parameters (*K_m_*, *V*_max_, *k*_cat,_ and *K_m_*/*k*_cat_) were determined by nonlinear regression fitting to the Michaelis–Menten equation, using GraphPad Prism version 8.0.2 for Windows (San Diego, CA, USA).

### 2.7. Modeling and Docking Analysis

The web-based protein structure prediction software SWISS-MODEL (https://swissmodel.expasy.org/, accessed on 15 July 2020) was used to predict the structure of Est19 and its E154D mutant. The best model was saved in PDB file format.

Ligand Docking tool in Maestro interface (Schrödinger Suite, LLC, New York, NY, USA) was used to conduct Est19, E154D variant, and *p*NPC6 docking. The Ligand docking was executed using the OPLS3 force field, with a threshold of 0 kcal/mol for rejecting minimized pose and the default settings for all other options. The candidate solution with the lowest energy was chosen for analysis. The structure figures were generated using PyMOL [30].

## 3. Results

### 3.1. Sequence Analysis and Multiple Alignment of Est19

The *est19* gene contained 954 bp of the open reading frame that encoded a precursor protein of 317 amino acids. The predicted molecular mass of the protein was 34.1 kDa. A signal sequence in Est19 was not detected. The protein BLAST analysis at NCBI showed that the first 100 amino acids of the sequence with the highest identity were all uncharacterized protein annotated from the bacterial genome sequence. According to the results of the BLAST in the ESTHER database, the protein was classified to the HSL family.

**Figure 1:**
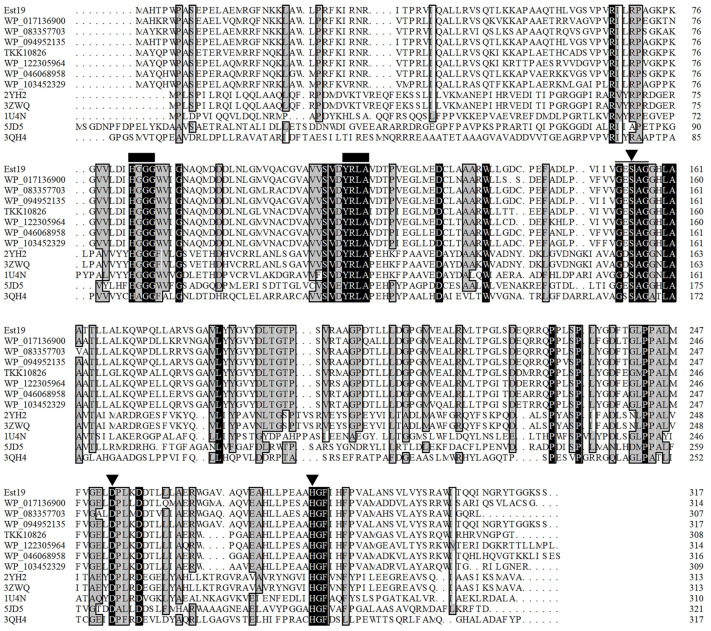
Sequence alignment of Est19 and members of the HSL family. Identical residues are shown in white on a black background, and similar residues (homology ≥ 75%) are on a grey background. Black triangles indicate residues forming the catalytic triad Ser, Asp, and His. Black squares indicate conserved motifs.

**Figure 2:**
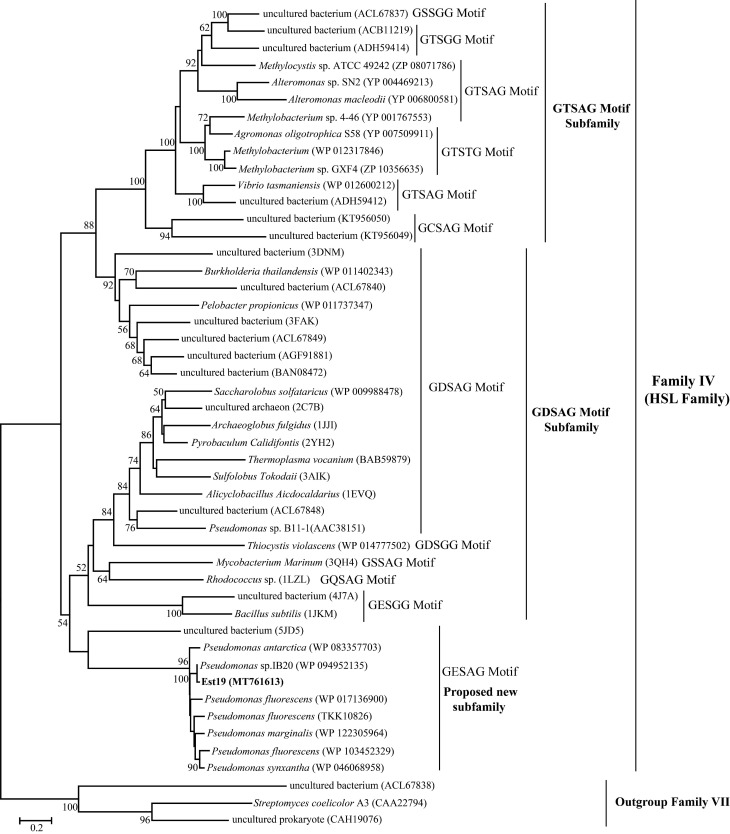
Phylogenetic analysis of Est19 and other members of the HSL family. The tree was constructed using the Maximum Likelihood method based on the JTT matrix-based model. Bootstrap analysis of 1000 replicates was conducted, and values above 50% are shown. The pentapeptide motif containing the catalytic Ser residue is shown for each sequence. Esterases from family VII were used as an outgroup.

The multiple-sequence alignment of Est19 with its homologous proteins revealed a catalytic triad composed of Ser^155^, Asp^253^, and His^282^ residues (Figure 1), and the catalytic Ser^155^ was located in a conserved motif GESAG. The HSL family characteristic sequence HGGG and the recently described YRLA motif [30] were also present in the amino acid sequences of Est19. Est19 and its closely related homologs formed an independent group other than the GDSAG and GTSAG subfamilies in the constructed phylogenetic tree, which indicated that Est19 represented a new subfamily of HSL enzymes (Figure 2).

### 3.2. Cloning, Expression, and Purification of Recombinant Est19

**Figure 3:**
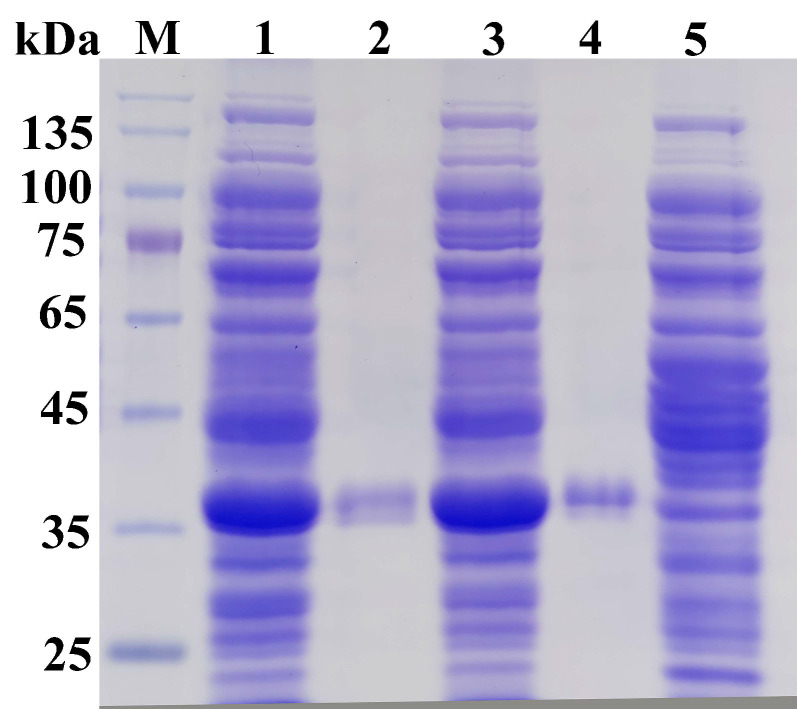
SDS-PAGE analysis of Est19 and E154D variant. Lane M, protein molecular mass marker; Lanes 1 and 3, total protein from *E. coli* with IPTG induction of Est19 and E154D variant, respectively; Lane 2, purified Est19; Lane 4, purified E154D variant; Lane 5, negative control, total protein from *E. coli* without IPTG induction.

The *est19* gene was successfully amplified by PCR, purified, and then ligated to the pET-22b vector. The vector construct was transformed into E. coli BL21 (DE3) for expression. The expressed protein contained a histidine tag that facilitated purification by Nickel-affinity chromatography. The SDS-PAGE showed that the molecular mass was ~35 kDa, consistent with the theoretical molecular mass (Figure 3). After dialysis, a relatively pure Est19 esterase was obtained. The sequencing of N-termini showed that the first amino acid residue Met^1^ was absent when expressed in *E.coli*.

### 3.3. Substrate Specificity and Kinetic Parameters

**Figure 4:**
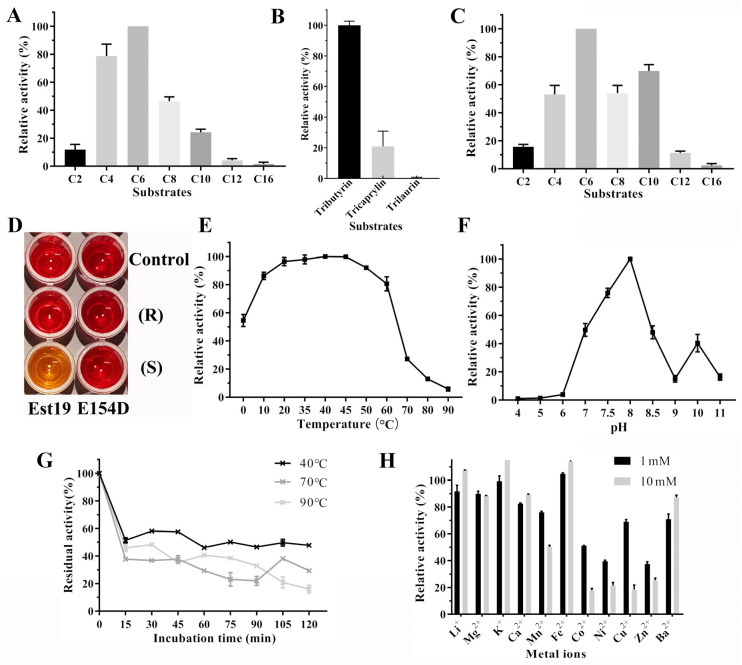
Biochemical characterization. (**A**) Substrate specificity of Est19. (**B**) Relative lipase activity of Est19 towards tributyrin, tricaprylin, and trilaurin. (**C**) Substrate specificity of E154D substitution. *p*NP esters (C2, C4, C6, C8, C10, C12, and C16) were used to determine the substrate specificity. (**D**) The hydrolysis activity of Est19 and E154D substitution toward methyl-L lactate or methyl-D lactate. The appearances of yellow color indicated the hydrolysis of the substrate. (**E**) Effect of temperature on the activity of Est19. (**F**) Effect of pH on the activity of Est19. (**G**) Thermostability assay. The enzyme was incubated at 40 °C, 70 °C, and 90 °C for different time and then residual activity was measured. (**H**) Effect of metal ions on the activity of Est19.

The substrate specificity of Est19 and its E154D variant was determined using p-nitrophenyl esters (pNP) with different acyl chain lengths. The purified enzymes hydrolyzed *p*NP esters up to 16 carbon atoms, with the highest level of hydrolytic activity toward *p*NPC6 (Figure 4A). Relatively low activity was observed toward *p*NPC12 and *p*NPC16, which had long-chain fatty acids. Moreover, as shown in Figure 4B, Est19 could hydrolyze tributyrin and tricaprylin, but not trilaurin. These data demonstrating preferential hydrolysis of short-chain acyl substrates indicated that Est19 behaved such as an esterase enzyme rather than a lipase. The E154D mutant had a higher relative activity toward *p*NPC10 than the wild enzyme (Figure 4C).

The kinetic parameter *K_m_*, *V*_max_, and *k*_cat_ values of Est19 for *p*NPC6 were 147 μM, 37,423 μM/min/mg, and 21.86 s^−1^, respectively. The catalytic efficiency (*k*_cat_/*K_m_*) of the esterase Est19 for *p*NPC6 was 148.68 s^−1^mM^−1^. When the residue Glu^154^ was mutated to Asp, *K_m_* increased while all the other parameters decreased (Table 1).

The stereo-specificity of the novel Est19 esterase and its E154D variant was investigated using the phenol red-based colorimetric method. In this method, the hydrolysis of chiral esters produces a visible color change. As shown in Figure 4D, the color of the solution containing methyl L-lactate changed significantly with Est19. No such color change was observed when methyl D-lactate was used as a substrate, indicating that Est19 can selectively hydrolyze methyl L-lactate, but not methyl D-lactate. In contrast, E154D variant could not hydrolyze either lactate forms (Figure 4D).

### 3.4. Effect of Temperature and pH on Esterase Activity and Stability

The effect of temperature and pH on esterase activity was measured with *p*NPC6 (Figure 4E,F). The results showed that the optimum temperature and pH were 40 °C and 8.0, respectively. Nearly 80% of Est19 maximal activity was retained over a wide temperature range from 10 to 60 °C. Remarkably, Est19 retained approximately 50% of its activity at 0 °C. However, its activity dropped sharply at 70 °C.

To further explore the enzymes’ thermal stability, the Est19 esterase was incubated at 40, 70, and 90 °C for different lengths of time (Figure 4G). The results showed that Est19 lost at least 40% of its activity after only 15 min at each of these temperatures. However, following the initial drop in activity (40 °C for 15 min), the residual activity of the esterase remained above 50% during a subsequent 2 h incubation. In contrast, treatment at 70 or 90 °C resulted in a rapid loss of esterase activity with little residual activity.

### 3.5. Effect of NaCl Concentration, Inhibitors, Metal Ions, and Organic Solvents on the Esterase Activity

As might be expected for an enzyme having a Ser-Asp-His catalytic triad, the serine modifying reagent PMSF remarkably inhibited the esterase activity of Est19 (Table 2). In contrast, the enzyme was relatively resistant to organosulfur redox reagents. The use of the thiol compound, DTT, slightly increased (30%) Est19 activity, whereas DMSO had little effect on its activity. The Est19 enzyme also tolerated relatively high NaCl concentrations. Roughly 87% of the Est19 activity was maintained when the salt concentration reached 1M. However, the enzyme precipitated when the NaCl reached concentrations >1.5 M.

The metal ion chelator, EDTA, also had no effect on Est19 activity, suggesting the enzyme did not require metal ions to function. The presence of millimolar concentrations of various metal ions was found to be inhibitory, however (Figure 4H). The use of Co^2+^, Ni^2+^, or Zn^2+^ at 1 mM in the concentration had a significant inhibitory effect on esterase activity, with residual enzyme activity ranging from 37% to 51.1%. Among them, the inhibitory effect of Co^2+^ increased significantly with increasing ion concentration to 10 mM (i.e., residual activity only 17.97%). Similar results were obtained with Cu^2+^, where the presence of 10 mM concentrations left only 21% of the initial Est19 activity.

The enzyme was also susceptible to inactivation using various denaturing agents. The use of both ionic (SDS) and non-ionic (TWEEN 80) detergents produced a marked inhibition (70–80%) of enzymatic activity. Similarly, nearly all the organic solvents tested inhibited Est19 substantially. In particular, the tolerance of Est19 to formaldehyde and ethanol was very low, with only 1.1–1.5% of activity remaining using 20% concentrations.

### 3.6. Modeling and Docking Analysis

**Figure 5:**
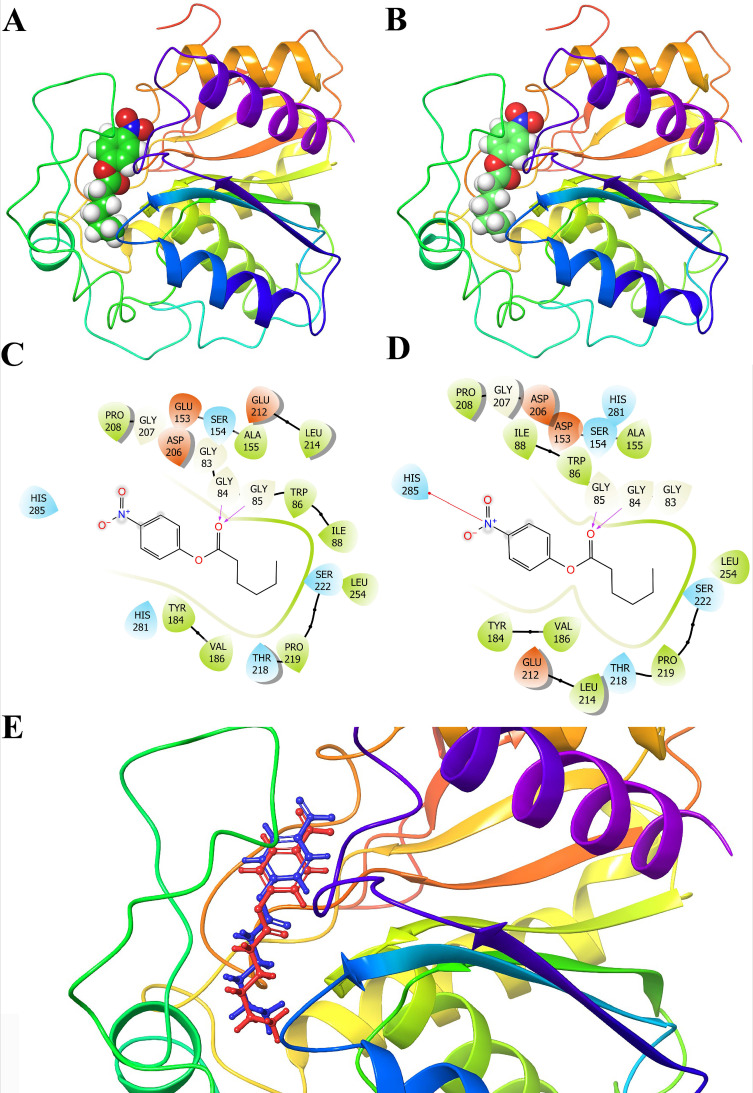
Structural analysis of the esterase. (**A**) Overall structure of Est19 modeled with *p*NPC6. (**B**) Overall structure of E154D substitution modeled with *p*NPC6. The structure revealed that they had a classical α/β hydrolase fold with 8 β-sheet and 6 α-helices. (**C**) H-bond formed between Gly^84^, Gly^85^ in the oxyanion hole of Est19 and carbonyl oxygen of *p*NPC6. (**D**) H-bond formed between Gly^84^, Gly^85^ residues in the oxyanion hole of E154D variant and carbonyl oxygen of *p*NPC6. (**E**) The detailed binding site. The *p*NPC6 modeled into Est19 and E154D variant was shown in red and blue, respectively. The distance between the two molecules was ~0.85 Å.

The structure of Est19 was constructed in SWISS-MODEL and an alpha/beta hydrolase enzyme MGS-MilE3 as a template (PDB 5JD5, 41.8% identity, 40.0% similarity, and 77.0% coverage), and the structure of the E154D mutant was constructed by Maestro based on the structure of Est19. The overall shape of the esterase was consistent with being a globular protein that is soluble and there were no detectable transmembrane regions. The predicted structure revealed that Est19 had a classical α/β hydrolase fold with eight β-sheet and six α-helices (Figure 5A,B).

In order to investigate the effect of the replacement of Glu^154^ by Asp, Est19, the E154D variant, and *p*NPC6, docking was conducted and the results indicated that an H-bond could form between amino acid Gly^84^, Gly^85^, and the carbonyl oxygen of *p*NPC6 (Figure 5C,D). The substrate pocket of the E154D variant changed compared to the wild one, which was assumed to happen because of the shorter side chain of Asp than Glu. It resulted in the substrate *p*NPC6 binding ~0.85 Å deeper into the pocket of the wild esterase than that of the mutant (Figure 5E).

## 4. Discussion

Here, we cloned an esterase gene *est19* from strain *P.* sp. E2-15. The resulting protein, esterase Est19, was then expressed and purified. Remarkably, Est19 retained 50% of its activity at 0 °C (Figure 4E). This was unique as two recently characterized esterases, GaDlh and EstA, lost most activity above and below their optimum temperature range [31,32]. Consequently, Est19 had a wide temperature tolerance, making it an excellent candidate for use in cold catalysis.

Bacterial lipolytic enzymes were divided into eight families [15]. So far, a considerable number of novel esterase has been identified, comprising 11 new families based on their biochemical and structural characters, phylogenetic analysis, and similarities of the conserved pentapeptide motif GXSXG [33]. Est19 belongs to the fourth family, namely, the HSL family, which was divided into two subfamilies, the GDSAG subfamily and the GTSAG subfamily. GESAG is the consensus motif of Family VII. However, Est19 and its homologous have a GESAG motif represent an independent branch of the HSL family, as shown in the phylogenetic tree (Figure 2).

The catalytic efficiency (*k*_cat_/*K_m_*) of the HSL family esterase varied a lot. For instance, the catalytic efficiency (*k*_cat_/*K_m_*) of Est19 for *p*NPC6 was 148.68 s^−1^mM^−1^, while that of esterase E25, a member of the GTSAG motif subfamily, for *p*NPC4 was 52 s^−1^mM^−1^ and that of esterase Clo1313, a member of the GDSAG motif subfamily, for *p*NPC4 was 285 s^−1^mM^−1^ [23,34]. It’s notable that by mutating Glu^154^ within the pentapeptide motif of Est19 to Asp, we found a dramatical decrease in the activity and catalytic efficiency (*k*_cat_/*K_m_*) (Table 1) of the enzyme. The introduction of the E154D substitution did not change the substrate preference for *p*NPC6. However, the catalytic activity of the variant for long-chain substrates *p*NPC10 and *p*NPC12 was enhanced. Because the side-chain of Glu had one more methylene group than that of Asp, the substrate pocket of E154D presented a deeper binding site compared to the wild Est19 (Figure 5). This deeper site may make it more difficult for smaller substrates to orient toward catalytic residues [35,36], while, conversely, enabling substrates with longer acyl chains have better access to increase the relative activity toward *p*NPC10 and *p*NPC12 substrates. Furthermore, the E154D variant resulted in the loss of activity toward methyl L-lactate, indicating that Glu^154^ may influence the enantioselectivity of Est19. The above data were consistent with Est19 being a new subfamily of the HSL family.

Most of the esterases previously reported in the HSL family displayed good activity under alkali conditions. The esterase EstD11 isolated from a hot spring metagenome exhibited peak activity at pH 8.0 [37], and the esterase PMGL2 obtained from a permafrost metagenomic library had a peak activity at pH 8.5 [38]. The optimum pH of Est19 was 8.0 (Figure 4F), indicating that it also functions effectively under alkali conditions, similar to most HSL family esterases previously reported.

Esterases have different response patterns of additives. The metal chelator EDTA had no effect on the activity of Est19, while DTT showed an activating effect. The other inhibitors PMSF, SDS, and TWEEN 80 remarkably inhibited enzyme Est19 (Table 2). The inhibition effect of PMSF suggests that a serine residue was involved in the catalytic site such as for other esterases [8,9]. The effect of organic reagents on enzyme activity was generally negative (Table 2). Especially, in the presence of 20% formaldehyde and ethanol, the residual enzyme activity was only 1.1–1.5%. In contrast, DMSO had little effect on the activity of Est19, as observed for other esterases [6,8]. Metal ions can have different effects on the activity of esterases. It has been reported that Ca^2+^, Co^2+^, Ba^2+^, and Mn^2+^ can increase the activity of esterases, while metal ions such as Cu^2+^, Zn^2+^, and Ni^2+^ have inhibitory effects [20]. The enzyme activity of esterase PMGL3 isolated from the frozen soil genome library was significantly inhibited by Zn^2+^, Ni^2+^, Cu^2+^, and Co^2+^ [39]. Similarly, the enzymatic activity of Est19 was inhibited by these same metal ions, especially by Cu^2+^, when the concentration reached 10 mM (Figure 4H). These observations supported the data on substrate specificity, indicating that Est19 behaved primarily as an esterase of the HSL family.

In conclusion, a novel esterase Est19 with the GESAG pentapeptide motif was identified from Antarctic bacteria *Pseudomonas* sp. E2-15. Biochemical and phylogenetic characteristics indicated that Est19 may represent a new subfamily of the HSL family. Furthermore, the ability of Est19 to retain a relatively high activity at low temperatures as well as its stereo-specificity makes Est19 a candidate esterase for cold and chiral catalyses.

## Figures and Tables

**Table 1:** Kinetic parameters of esterase Est19 and E154D variant for pNPC6 ^a^.

Enzyme	*K_m_* (μM)	*V*_max_ (μM/min/mg)	*k*_cat_ (s^−1^)	*k*_cat_/*K_m_* (s^−1^mM^−1^)
Est19	147 ± 28	37423 ± 2478	21.86 ± 1.45	148.68
E154D mutant	187 ± 28	25671 ± 1321	14.99 ± 0.77	80.14

^a^ Reactions were conducted in triplicate at 40 °C in 50 mM Tris-HCl buffer (pH 8.0) using *p*NPC6 as substrate. Results were showed as the mean ± SD.

**Table 2:** Relative activity percentage in the presence of additives.

Additive		Residual Activity	
NaCl	NaCl	0.5 M	1 M
		87.52 ± 5.50	86.50 ± 6.04
Inhibitor		1 mM	10 mM
	EDTA	108.59 ± 5.68	102.75 ± 1.93
	PMSF	75.52 ± 2.76	13.33 ± 2.64
	DTT	128.93 ± 7.49	132.97 ± 3.19
		1%	5%
	SDS	18.71 ± 3.46	5.50 ± 1.24
	TWEEN 80	30.87 ± 1.98	9.81 ± 1.06
Organic regent		20% (*v*/*v*)	40% (*v*/*v*)
	Methanol	23.21 ± 2.87	27.99 ± 2.30
	Formaldehyde	1.15 ± 0.05	0
	Ethanol	1.19 ± 0.22	0
	Acetonitrile	28.74 ± 0.73	9.97 ± 0.04
	Acetone	33.41 ± 0.22	17.18 ± 0.32
	Isopropanol	33.66 ± 1.31	18.37 ± 0.33
	DMSO	92.94 ± 0.50	45.54 ± 0.09

## Data Availability

Data supporting reported results are contained within the article.
